# Incidence and costs of hospitalisation due to acute respiratory infection in adults aged over 50 years in Jiangsu, China in 2019–23: a real-world medical database analysis of 0.2 million episodes

**DOI:** 10.7189/jogh.15.04116

**Published:** 2025-03-14

**Authors:** Xiaoyu Xu, Ling Guo, Xiao Li, Xin Wang, You Li

**Affiliations:** 1 Department of Epidemiology, School of Public Health, Key Laboratory of Public Health Safety and Emergency Prevention and Control Technology of Higher Education Institutions in Jiangsu Province, Nanjing Medical University, Nanjing, China; 2 Centre for Health Economics Research and Modelling Infectious Diseases (CHERMID), University of Antwerp, Antwerp, Belgium; 3 Department of Biostatistics, National Vaccine Innovation Platform, School of Public Health, Nanjing Medical University, Nanjing, China; 4 Centre for Global Health, Usher Institute, University of Edinburgh, Edinburgh, UK; 5 Changzhou Third People’s Hospital, Changzhou Medical Centre, Nanjing Medical University, Changzhou, China

## Abstract

**Background:**

Acute respiratory infection (ARI) poses a significant public health challenge worldwide, particularly among older adults. However, the disease and economic burden of ARI among older adults in China remained sparse. We aimed to estimate the incidence rate and medical cost of hospitalisation due to ARI among adults aged ≥50 years in Jiangsu Province, China.

**Methods:**

We analysed medical records of hospitalised episodes due to ARI from January 2019 to May 2023 from a regionally representative medical database. We estimated hospitalisation rates and the proportion of severe cases (intensive care unit admission, mechanical ventilation, or death) by sex, age group, and time period. Total direct medical cost and out-of-pocket cost were estimated in CNY. We analysed factors influencing total costs using a multivariate linear regression model. We further compared the proportion of severe cases and medical cost between those with and without selected comorbidities.

**Results:**

A total of 209 632 episodes of ARI hospitalisation were included. Over the study period, annualised ARI hospitalisation rate ranged from 1.07 to 1.83 per 1000 person-years, and varied by age, sex and region. Severe cases accounted for 6.5–10.3%. The median total direct medical cost was CNY 9027 (interquartile range (IQR) = 6118–14 886), of which 22% (IQR = 7–41) was out-of-pocket. Rural residents born a substantial out-of-pocket cost, which was even higher than their average monthly disposable income. Patients with certain comorbidities had higher medical costs despite having a similar or even lower proportion of severe cases.

**Conclusions:**

By analysing a large regionally representative medical database, we helped address the knowledge gap in the burden and cost of ARI hospitalisation in China. While highlighting the overall substantial disease burden and cost of ARI, we identified important factors such as age, sex, region, and comorbidity that influence the disease burden and cost of ARI.

Acute respiratory infection (ARI), including acute upper respiratory and lower respiratory infections, poses a significant public health challenge worldwide. Older adults are particularly vulnerable to ARI due to immunosenescence and increased prevalence of comorbidities [1]. The Global Burden of Disease 2021 study estimated that non-COVID ARI caused 2.18 million deaths globally, of which 68% were people aged ≥50 years [2]. ARI is also responsible for a substantial economic burden; a recently published systematic review estimated that the overall mean cost of ARI management in older adults was EUR 17 804 across 20 countries globally [3].

As one of the most populated countries in the world, China bears a substantial disease burden of ARI, particularly for older adults, which is expected to further increase due to the ageing population. With the growing availability of vaccines for common non-COVID ARI aetiologies, such as the influenza vaccine, pneumococcal vaccine, and most recently respiratory syncytial virus vaccine that received licensure in Hong Kong, China [4], it is important to understand the incidence rate and medical cost of ARI for estimating the impact of implementation of ARI-related vaccination.

As there is no nationwide surveillance of ARI in China, evidence on the incidence rate and medical cost of ARI remains sparse, and is primarily limited to single centre or city level. An early nationwide study across 23 provinces of China estimated that in 2016, the incidence rate of community-acquired pneumonia (a form of acute lower respiratory infection) among urban residents who were enrolled in the Chinese urban basic medical insurance was nine out of 1000; the estimated incidence rates rose over increased age among adults [5]. Another study based on the China Kadoorie Biobank reported an increasing trend in pneumonia hospitalisation rate among adults aged >30 years between 2009–17 [6]. However, both studies were conducted before the COVID-19 pandemic, which was known to have substantially changed the epidemiology of multiple infectious diseases in China and beyond. Regarding the cost of ARI management, our previously published systematic review estimated that the median (MD) cost of ARI management in older adults was USD 3263 per inpatient episode in China [7]. However, this review was based primarily on single-hospital studies, having limited representativeness of the population.

To help further address the knowledge gaps in the incidence rate and medical cost of ARI in older adults, we conducted a retrospective medical database analysis of hospitalisation due to non-COVID ARI among adults aged ≥50 years in Jiangsu Province, China, spanning both pre- and post-COVID-19 pandemic periods.

## METHODS

### Data source

We retrieved de-identified individual-patient-level medical records from a regional medical database, covering more than 200 hospitals (over 95% coverage of grade-III hospitals, the most common grade of hospitals with beds in Jiangsu Province) from all the 13 municipalities of Jiangsu Province. More details on the data governance, storage, and processing can be found elsewhere [8].

### Study population

For this analysis, we extracted the medical records of hospitalised cases aged ≥50 years due to non-COVID-19 ARI from 1 January 2019 to 3 May 2023, identified by the primary diagnosis at discharge according to the international classification of diseases (ICD-10) codes (Table S1 in the **Online Supplementary Document**). We excluded individuals whose place of residence was outside Jiangsu Province and duplicate records. Moreover, there was an unprecedented major COVID-19 epidemic during the last three weeks of 2022 and the first three weeks of 2023, associated with the full relaxation of all non-pharmaceutical interventions; some hospitals did not differentiate COVID from non-COVID cases during this period while COVID cases dominated during this period. Therefore, we excluded the records during 11 December 2022 to 21 January 2023 to avoid counting COVID cases in the analysis (this period was re-included in the sensitivity analysis).

### Data collection

For the study population, the medical records retrieved consisted of basic characteristics, an inpatient record summary, and a cost summary. Basic characteristics included the patient’s date of birth, sex, and residential address (specific to county level), as well as the grade of hospital. In China, there are three grades of hospitals, with the highest grade (*i.e.* grade III) having the highest standard of care and capacity. Grade I hospitals are community-based hospitals and usually do not have hospital beds.

Inpatient record summary included dates of admission and discharge, source of admission (*e.g.* from emergency department, outpatient, and so on), primary and secondary diagnoses coded by ICD-10 (a patient could have only one primary diagnosis and multiple secondary diagnoses; in this study, we could only include the first two secondary diagnoses), use of intensive care unit (ICU), use of mechanical ventilation, and clinical outcome at discharge (*e.g.* fully recovered, improved, not improved, dead, and so on). We used secondary diagnosis to identify patients with selected comorbidities that were deemed to be risk factors for ARI hospitalisation, including chronic obstructive pulmonary disease (COPD), asthma, ischemic heart disease (IHD), stroke, diabetes, chronic kidney disease (CKD), and chronic liver disease [9] (Table S2 in the **Online Supplementary Document**).

Cost summary included the total and category-specific medical costs from a health care system perspective, out-of-pocket (OOP) costs, and health insurance category of the patient (the three common health insurances in China are the urban employee basic medical insurance (UEBMI), which cover employed urban residents, the urban resident basic medical insurance (URBMI), which cover unemployed urban residents (including students), and the New Rural Cooperative Medical Scheme (NCMS), which cover rural residents). As the cost of antibiotics was available in the cost summary data as a separate cost category, we used this cost category to indicate whether a patient received antibiotic treatment during admission. For cost analysis, individuals with total hospitalisation costs falling in the extreme 0.25% at either end of the cost distribution were excluded.

### Statistical analysis

For all statistical analyses, we reported the following time periods separately, January 2019 to December 2019, January 2020 to December 2020, January 2021 to December 2021, and January 2022 to December 2022. As data were available only for the first five months of 2023, to allow comparison of incidence rate, we used the last available 12 months for reporting, which was June 2022 to May 2023.

**Figure 1:**
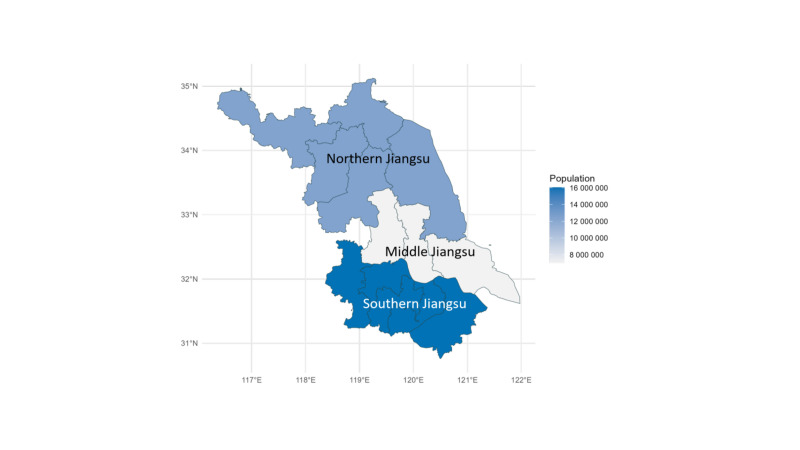
The map of study setting with information of population aged ≥50 years in 2022 (total population in Jiangsu Province: 35.51 million). Northern Jiangsu included five municipalities: Xuzhou, Lianyungang, Suqian, Huaian, and Yancheng. Middle Jiangsu included three municipalities: Yangzhou, Taizhou, and Nantong. Southern Jiangsu included five municipalities: Nanjing, Suzhou, Wuxi, Changzhou, and Zhenjiang.

We estimated the incidence rate of ARI hospitalisation, overall, by age and sex, and by region (**Figure 1**). Numerators were the number of ARI hospitalisations in the corresponding categories and population denominators were extracted from Jiangsu Statistical Yearbook [10–13]. We also estimated the proportion of severe ARI cases, defined as the use of mechanical ventilation, admission to ICU, or death.

We estimated MD and interquartile range (IQR), and mean (x̄) and standard deviation (SD) of the total costs. OOP cost and its proportion in total cost were estimated and reported as MD and IQR. All estimates above were reported overall, and by different categories, including age group, sex, hospital grade, disease category (by ICD-10), time period, ICU admission, clinical outcome, source of admission, use of ventilation, use of antibiotics, pathogen-specific ICD diagnosis (bacterial/viral infections), health insurance, and region. To further explore the factors influencing medical costs associated with ARI, we conducted a multi-variate linear regression analysis of total costs with following independent variables: age group, sex, comorbidities, length of stay, region, type of health insurance, and hospital grade. We also explored the correlation between total cost and gross domestic product (GDP) per capita at the municipality level. We reported the medical cost broken down by category of costs, including nursing cost, diagnosis cost (including laboratory diagnosis cost, imaging diagnosis cost, and clinical diagnosis cost), treatment cost, medicine cost, and consumable cost. Moreover, we compared the proportion of severe cases as well as medical cost between patients with selected comorbidities and those without comorbidities.

We initially recorded all costs in CNY for the year of discharge, and then adjusted to the year 2023 value according to the annual consumer price index for health care from the National Bureau of Statistics of China [14]. Discounting of costs was not applied in this study as long-term economic effectiveness was not involved. All analyses were conducted using *R*, version 4.3.1 (R Core Team, Vienna, Austria).

## RESULTS

After applying the inclusion and exclusion criteria, we included a total of 209 632 episodes in this study; the number of episodes included for the cost analysis was smaller (n = 207 657) due to missing cost data (**Table 1**; Figure S1 in the **Online Supplementary Document**). The age of the patients was x̄ = 70.78 years (SD = 11.65), and the most (58.0%, n = 121 439) were male. The majority (93.25%, n = 195 412) received medical services from grade III hospitals. Pneumonia and influenza were the most common diagnosis category, accounting for 80.8% (n = 169 322) of all diagnoses. Overall, the three regions, namely northern, middle, and southern Jiangsu (**Figure 1**), accounted for approximately 35%, 20%, and 45% of the population aged ≥50 years, and accounted for approximately 28%, 19%, and 53% of the ARI hospitalisation episodes (**Table 1**).

**Table 1:** Basic characteristics of non-COVID episodes included in the study*

Characteristics	Episodes included in the burden analysis (n = 209 632)	Episodes included in the cost analysis (n = 207 657)
Sex		
*Male*	121 439 (58.0)	120 199 (57.9)
*Female*	88 085 (42.0)	87 350 (42.1)
Age in years, x̄ (SD)	70.78 (11.65)	70.78 (11.64)
Age group in years		
*50–60*	47 392 (22.6)	46 871 (22.6)
*60–70*	54 044 (25.8)	53 561 (25.8)
*70–80*	56 434 (26.9)	55 985 (27.0)
*≥80*	51 762 (24.7)	51 240 (24.7)
Grade of the hospital		
*Grade III general hospital*	176 043 (84.0)	174 793 (84.2)
*Grade III specialty hospital*	19 369 (9.2)	18 693 (9.0)
*Grade II hospital*	14 118 (6.7)	14 071 (6.8)
*Other medical services*	102 (0.05)	100 (0.05)
Disease category by ICD-10		
*Pneumonia and influenza*	169 322 (80.8)	167 953 (80.9)
*AURI*	14 361 (6.9)	14 133 (6.8)
*Other ALRI*	25 949 (12.4)	25 571 (12.3)
Time period		
*January–December 2019*	33 948 (12.9)	33 737 (16.2)
*January–December 2020*	35 792 (13.7)	35 472 (17.1)
*January–December 2021*	50 142 (19.1)	49 653 (23.9)
*January–December 2022*	55 695 (26.6)	55 193 (26.6)
*June 2022–May 2023*	65 159 (31.1)	64 449 (31.0)
ICU admission		
*Yes*	14 853 (7.1)	14 457 (7.0)
*No*	194 779 (92.9)	193 200 (93.0)
Clinical outcome		
*Fully recovered*	9692 (4.6)	9638 (4.6)
*Improved*	186 375 (88.9)	184 884 (89.0)
*Not improved*	9031 (4.3)	8783 (4.2)
*Died (in hospital)*	2442 (1.2)	2313 (1.1)
*Unknown*	2092 (1.0)	2039 (1.0)
Ventilation use time in hours		
*0*	206 151 (98.3)	204 342 (98.4)
*1–96*	1247 (0.6)	1238 (0.6)
*>96*	2234 (1.1)	2077 (1.0)
Antibiotics use		
*No*	50 656 (24.7)	49 377 (24.3)
*Yes*	154 595 (75.3)	153 926 (75.7)
Region†		
*Northern Jiangsu*	57 917 (27.7)	57 608 (27.8)
*Middle Jiangsu*	40 291 (19.3)	40 137 (19.4)
*Southern Jiangsu*	110 807 (53.0)	109 317 (52.8)
*Missing*	617 (0.3)	595 (0.3)

ALRI – acute lower respiratory infection, AURI – acute upper respiratory infection, ICD – international classification of diseases, ICU – intensive care unit, SD – standard deviation, x̄ – mean

*Presented as n (%) unless specified otherwise.

†Northern Jiangsu included five municipalities: Xuzhou, Lianyungang, Suqian, Huaian, and Yancheng. Middle Jiangsu included three municipalities: Yangzhou, Taizhou, and Nantong. Southern Jiangsu included five municipalities: Nanjing, Suzhou, Wuxi, Changzhou, and Zhenjiang.

### Hospitalisation burden

The hospitalisation rate of ARI in older adults aged ≥50 years varied by time period, ranging from 1.07 (95% confidence interval (CI) = 1.05–1.08) per 1000 person-years during January to December of 2019 to 1.83 (95% CI = 1.82–1.85) per 1000 person-years during June 2022 to May 2023. Southern Jiangsu had relatively higher hospitalisation rates over the study period while Northern Jiangsu had relatively lower hospitalisation rates (**Table 2**). For all regions across the study periods, hospitalisation rate increased with age (Figure S2 in the **Online Supplementary Document**). The hospitalisation rate ratio between those aged ≥80 years and those aged 50–60 years ranged from 4.8 to 6.6 per 1000 person-years across time periods. Within a year, hospitalisation generally peaked during the winter and spring seasons; the only exception was during the major COVID epidemic period during winter of 2022–23; the peak was not observed until March 2023 (Figure S3 in the **Online Supplementary Document**). Hospitalisation rates were higher in males than in females across all age groups of ≥60 years, with the most pronounced difference observed in the age group of ≥80 years. By contrast, the hospitalisation rate in the age group of 50–60 years was higher among females than in males during some time periods (**Table 2**). Sensitivity analysis that included the local COVID-19 epidemic period yielded a higher hospitalisation rate during the corresponding time period but demonstrated similar age-, sex- and region-specific patterns to the main analysis (Table S3–5 in the **Online Supplementary Document**).

**Table 2:** Hospitalisation rates of non-COVID ARI among those aged ≥50 y in Jiangsu, China

	Hospitalisation rate, per 1000 person-years (95% CI)				
**Category**	**January–December 2019**	**January–December 2020**	**January–December 2021**	**January–December 2022**	**June 2022–May 2023**
All population	1.07 (1.05–1.08)	1.09 (1.08–1.10)	1.46 (1.45–1.47)	1.57 (1.56–1.58)	1.83 (1.82–1.85)
Age group 50–60 y					
*All*	0.64 (0.63–0.65)	0.57 (0.56–0.59)	0.78 (0.77–0.79)	0.75 (0.74–0.76)	0.86 (0.85–0.87)
*Male*	0.57 (0.56–0.59)	0.58 (0.56–0.60)	0.74 (0.73–0.76)	0.75 (0.73–0.77)	0.85 (0.83–0.87)
*Female*	0.70 (0.68–0.72)	0.57 (0.55–0.59)	0.81 (0.79–0.83)	0.75 (0.73–0.77)	0.87 (0.85–0.89)
Age group 60–70 y					
*All*	0.93 (0.91–0.95)	0.96 (0.94–0.98)	1.34 (1.32–1.36)	1.30 (1.27–1.32)	1.52 (1.50–1.55)
*Male*	1.02 (0.99–1.04)	1.14 (1.11–1.17)	1.59 (1.56–1.63)	1.54 (1.51–1.58)	1.77 (1.73–1.80)
*Female*	0.85 (0.82–0.87)	0.78 (0.75–0.80)	1.08 (1.05–1.11)	1.05 (1.02–1.08)	1.27 (1.24–1.30)
Age group 70–80 y					
*All*	1.41 (1.38–1.45)	1.59 (1.56–1.62)	2.12 (2.08–2.16)	2.36 (2.32–2.39)	2.84 (2.80–2.88)
*Male*	1.72 (1.68–1.77)	2.07 (2.02–2.13)	2.76 (2.70–2.82)	3.08 (3.02–3.14)	3.69 (3.63–3.76)
*Female*	1.12 (1.08–1.16)	1.14 (1.10–1.18)	1.51 (1.47–1.56)	1.68 (1.63–1.72)	2.03 (1.99–2.08)
Age group ≥80 y					
*All*	3.09 (3.02–3.16)	3.16 (3.10–3.23)	4.07 (4.00–4.14)	4.91 (4.83–4.98)	5.62 (5.53–5.70)
*Male*	4.32 (4.20–4.45)	4.77 (4.64–4.90)	6.14 (6.00–6.28)	7.40 (7.25–7.55)	8.22 (8.07–8.38)
*Female*	2.20 (2.12–2.28)	2.03 (1.96–2.10)	2.56 (2.49–2.64)	3.06 (2.98–3.14)	3.67 (3.58–3.76)
Region*†					
*Northern Jiangsu*	0.85 (0.83–0.86)	0.81 (0.80–0.83)	1.07 (1.05–1.08)	1.21 (1.19–1.23)	1.59 (1.56–1.61)
*Middle Jiangsu*	0.72 (0.70–0.74)	1.03 (1.00–1.05)	1.41 (1.38–1.44)	1.74 (1.71–1.77)	1.99 (1.95–2.02)
*Southern Jiangsu*	1.37 (1.35–1.39)	1.33 (1.31–1.34)	1.79 (1.77–1.81)	1.76 (1.74–1.78)	1.95 (1.93–1.97)

ARI – acute respiratory infection, CI – confidence interval

*Northern Jiangsu included five municipalities: Xuzhou, Lianyungang, Suqian, Huaian, and Yancheng. Middle Jiangsu included three municipalities: Yangzhou, Taizhou, and Nantong. Southern Jiangsu included five municipalities: Nanjing, Suzhou, Wuxi, Changzhou, and Zhenjiang.

†The hospitalisation rates of different regions were standardised by age and gender distribution of population census data in Jiangsu Province.

Among hospitalised cases, 6.5–10.3% received mechanical ventilation, were admitted to ICU, or died during hospital stay (defined as severe cases). The proportion of severe cases was higher in males than females and increased with age. (Table S6 in the **Online Supplementary Document**).

### Hospitalisation cost

The MD total direct medical cost per episode was CNY 9027 (IQR = 6118–14 886; x̄ = 16 488, SD = 27 120). The MD OOP direct medical cost per episode was CNY 2028 (IQR = 601–4072), accounting for 22% (IQR = 7–41) of total direct medical cost. The MD length of stay was nine days (IQR = 6–12) (**Table 3**; Table S7 in the **Online Supplementary Document**).

**Table 3:** Summary of direct medical cost per episode of non-COVID ARI hospitalisation in CNY of 2023*

Category	n	Total cost	OOP cost	Proportion of OOP cost in %	Length of stay
All population	207 657	9027 (6118–14 886)	2028 (601–4072)	22 (7–41)	9 (6–12)
Age group in years					
*50–60*	46 871	7354 (5130–10 690)	1780 (434–3395)	25 (7–44)	8 (6–11)
*60–70*	53 561	8485 (5876–12 859)	1931 (561–3794)	23 (8–42)	8 (6–12)
*70–80*	55 985	9582 (6578–15 463)	2186 (720–4371)	22 (9–41)	9 (6–13)
*≥80*	51 240	11 424 (7375–21 266)	2259 (682–4813)	19 (6–38)	10 (7–14)
Sex					
*Male*	120 199	9722 (6432–16 612)	2009 (548–4302)	20 (6–39)	9 (6–13)
*Female*	87 350	8277 (5774–12 295)	2052 (684–3829)	25 (9–44)	8 (6–11)
Grade of hospital					
*Grade III general hospital*	174 793	9145 (6221–14 746)	2069 (611–4148)	22 (7–42)	9 (6–12)
*Grade III specialty hospital*	18 693	10 013 (6828–15 571)	2003 (580–4172)	18 (6–32)	10 (7–14)
*Grade II hospital*	14 071	6736 (4726–9922)	1645 (551–3064)	25 (9–43)	7 (5–10)
*Other medical services*	100	3296 (2660–4375)	569 (184–839)	17 (4–25)	7 (6–8)
Disease category					
*Pneumonia and influenza*	167 953	9817 (6755–16 083)	2239 (711–4520)	22 (8–41)	9 (7–13)
*AURI*	14 133	4615 (3245–6824)	1217 (245–2217)	26 (6–49)	6 (4–8)
*Other ALRI*	25 571	7183 (5092–10 135)	1525 (385–2819)	22 (5–41)	7 (5–10)
Time period					
*January–December 2019*	33 737	8707 (5826–13 670)	1838 (362–3722)	21 (5–41)	8 (6–12)
*January–December 2020*	35 472	9332 (6200–15 426)	1865 (471–3923)	20 (6–39)	9 (6–13)
*January–December 2021*	49 653	9486 (6376–15 571)	2069 (635–4255)	21 (7–39)	9 (6–13)
*January–December 2022*	55 193	8928 (6095–14 550)	2185 (805–4332)	23 (10–44)	8 (6–12)
*June 2022–May 2023*	64 449	8748 (6063–13 726)	2121 (750–4084)	23 (9–43)	8 (6–12)
ICU admission					
*Yes*	14 457	32 634 (9610–76 655)	4899 (1344–14 964)	19 (8–37)	11 (6–19)
*No*	193 200	8767 (6022–13 419)	1957 (576–3800)	22 (7–41)	9 (6–12)
Clinical outcome					
*Fully recovered*	9638	5895 (3985–9288)	1336 (162–2673)	22 (3–43)	7 (5–10)
*Improved*	184 884	9003 (6224–13 920)	2022 (610–3943)	22 (7–41)	9 (6–12)
*Not improved*	8783	19 454 (7832–54 483)	3721 (1173–11 106)	22 (10–43)	7 (3–14)
*Died*	2313	44 114 (16 428–96 283)	4613 (448–15 517)	16 (2–27)	10 (4–19)
*Unknown*	2039	8362 (4944–18 153)	2510 (762–6132)	26 (10–93)	7 (3–12)
Source of admission					
*Emergency*	67 599	10 371 (6647–19 845)	2452 (957–5220)	22 (10–41)	9 (6–13)
*Outpatient*	137 562	8563 (5928–12 945)	1845 (455–3627)	22 (6–41)	8 (6–12)
*Transfer*	1657	8169 (5499–12 826)	2076 (613–4704)	24 (9–51)	8 (6–12)
*Other*	839	8542 (5588–15 267)	2414 (728–5413)	19 (8–100)	9 (6–13)
Ventilation use time in hours					
*0*	204 342	8928 (6076–14 113)	1998 (582–3960)	22 (7–41)	9 (6–12)
*1–96*	1238	27 703 (13 329–49 656)	6690 (2598–14 136)	26 (14–43)	6 (2–13)
*>96*	2077	93 860 (58 133–149 067)	16 847 (7787–32 470)	19 (11–31)	19 (12–28)
Antibiotics use					
*Yes*	153 926	9279 (6351–14 845)	2209 (848–4281)	23 (10–42)	9 (6–13)
*No*	49 377	8156 (5390–13 356)	1431 (29–3222)	19 (0–41)	8 (6–12)
Pathogen-specific ICD diagnosis					
*Bacterial infection*	1448	24 532 (13 227–49 066)	3479 (1246–7497)	15 (5–26)	18 (11–25)
*Viral infection*	1156	7435 (4970–11 637)	2059 (898–3879)	29 (12–47)	7 (5–10)
Health insurance					
*UEBMI*	104 872	9525 (6479–15 387)	1627 (364–3181)	17 (4–27)	9 (6–13)
*URBMI*	68 858	8185 (5672–12 324)	2439 (933–4357)	34 (12–49)	8 (6–11)
*NCMS*	8323	7561 (5263–11 166)	2565 (1230–4144)	40 (18–48)	8 (6–11)
*Other*	25 604	10 674 (6481–20 326)	3246 (719–8385)	29 (6–100)	9 (6–13)
Region†					
*Northern Jiangsu*	57 608	7242 (5044–10 575)	1943 (745–3436)	28 (10–46)	8 (6–11)
*Middle Jiangsu*	40 137	9054 (6190–13 959)	1928 (522–4333)	21 (6–44)	9 (6–12)
*Southern Jiangsu*	109 317	10 257 (6930–17 144)	2116 (502–4447)	20 (5–36)	9 (7–13)

ALRI – acute lower respiratory infection, ARI – acute respiratory infection, AURI – acute upper respiratory infection, IQR – interquartile range, MD – median, NCMS – new rural cooperative medical scheme, OOP – out of pocket, UEBMI – urban employee basic medical insurance, URBMI – urban resident basic medical insurance

*Presented as MD (IQR).

†Northern Jiangsu included five municipalities: Xuzhou, Lianyungang, Suqian, Huaian, and Yancheng. Middle Jiangsu included three municipalities: Yangzhou, Taizhou, and Nantong. Southern Jiangsu included five municipalities: Nanjing, Suzhou, Wuxi, Changzhou, and Zhenjiang.

Overall, no clear trends over the study periods were observed in the total direct medical cost or in the MD length of stay. The MD direct medical cost was lowest in the youngest age group (50–60 years, CNY 7354) and was highest in the oldest age group (≥80 years, CNY 11 424). The MD direct medical cost was higher among males than females, and was higher in grade III hospitals than non-grade III hospitals. Those who were admitted to ICU, received mechanical ventilation or died in hospitals had much higher direct medical cost, with the MD cost being over CNY 27 000 (approximately three times higher than the MD cost among all non-COVID ARI episodes). Among those with pathogen-specific ICD diagnosis (n = 2604), the MD cost was much higher in those with bacterial infections than those with viral infections. While those covered by UEBMI tended to have higher total costs, the OOP cost was lowest compared to those covered by other health insurances (**Table 3**). Besides, regional variations were noted in the median direct medical cost; the GDP per capita at the municipality level was positively correlated with the direct medical cost (Pearson *r* = 0.907; *P* < 0.001) (Figure S4 in the **Online Supplementary Document**).

**Figure 2:**
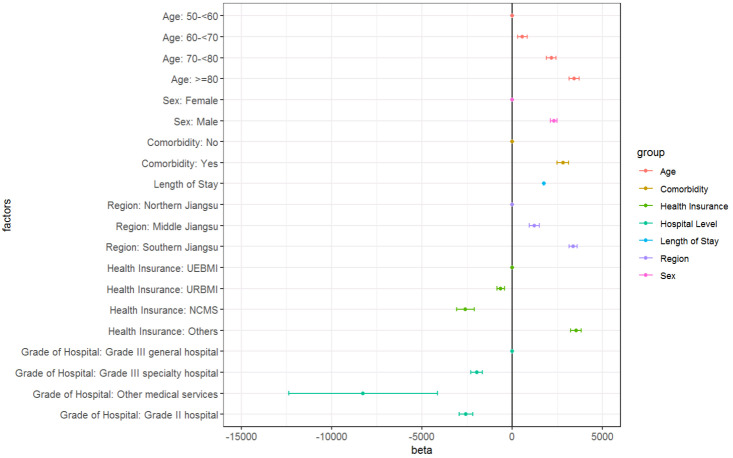
Multi-variate linear regression analysis of total direct medical costs of ARI. ARI – acute respiratory infection, NCMS – new rural cooperative medical scheme, UEBMI – urban employee basic medical insurance, URBMI – urban resident basic medical insurance.

Multi-variate regression analysis showed consistent results. Older age, males, comorbidities, longer length of stays, southern region of Jiangsu Province, higher grade of hospitals and being covered by UEBMI were associated with higher direct medical costs (**Figure 2**).

Of all components of direct medical cost, diagnosis cost (MD = 37, IQR = 26–49) and medication cost (MD = 37; IQR = 26–47) jointly accounted for over 70% of the cost. Laboratory diagnosis cost accounted for the most in the diagnosis cost, with a median of CNY 2401 (IQR = 1537–3620) (Table S8 in the **Online Supplementary Document**).

### Burden and cost by comorbidity status

We identified 2099–17 358 non-COVID episodes with any documented comorbidities of COPD, asthma, IHD, stroke, diabetes, CKD, and chronic liver disease, and 18 482 episodes with no documented comorbidities. Compared to those with no comorbidities, patients with COPD, asthma, IHD, diabetes, or chronic liver disease had lower proportion of severe cases, and patients with stroke or CKD had comparable proportion of severe cases. By contrast, patients with any of those seven comorbidities had higher MD direct medical costs (ranging from CNY 8601 of chronic liver disease to CNY 10 102 of CKD) than patients without any comorbidities (CNY 6085) (Table S9 in the **Online Supplementary Document**).

## DISCUSSION

In this retrospective study, we estimated hospitalisation burden and direct medical costs of non-COVID ARI episodes among older adults aged ≥50 years in Jiangsu, China during January 2019 to May 2023, using a large regionally representative medical database. We highlighted the substantial disease burden and economic burden of ARI in the study population, which further varied by age, sex, region, and comorbidity status.

We demonstrated increased hospitalisation rates of ARI over the study period, ranging from 1.07 (95% CI = 1.05–1.08) per 1000 person-years during January to December of 2019 to 1.83 (95% CI = 1.82–1.85) per 1000 person-years during June 2022 to May 2023. A similar secular trend was previously reported in a large cohort study in China (China Kadoorie Biobank), which estimated an annual increase of 14.3% after adjusting for the aging effect of the cohort and prevalence of comorbidities [6]. While admitting that the COVID-19 pandemic and its associated non-pharmaceutical interventions could drive the hospitalisation rate, we speculate that the increased hospitalised rate was more likely due to the steadily increased health care service capacity. According to Jiangsu Provincial Health Commission, the number of hospital beds increased from 516 000 in 2019 to 579 000 by the end of 2023 (representing approximately 12% increase) [15]. Considering that the MD length of stay for an ARI episode was <10 days (as shown in this study), one additional hospital bed in the respiratory ward could be used by at least 12 ARI patients during a regular four-month respiratory season. In addition to health care service capacity, health care seeking behaviours and admission criteria might have changed over the course of the pandemic and could explain the rise in the hospitalisation rate.

In this study, we focused only on hospitalised cases that were clinically diagnosed as being due to ARI (*i.e.* as the primary diagnosis), by excluding cases that had ARI diagnosis in the secondary diagnosis field. We believe that such restriction is important for understanding the number of directly avertable hospitalisation by targeted prevention against ARI (such as vaccination), and for understanding the medical cost that was attributable to ARI. As expected, the hospitalisation rate estimates in this study were generally lower than previous studies that did not restrict to ARI as the main diagnosis [5,6].

Similar to the global-level estimates [16], we found that adult ARI hospitalisation rate and proportion of severe cases increased over age and were generally higher in males than in females. The only exception was in the age group of 50–60 years where females had comparable or even higher hospitalisation rates than males; interestingly, similar patterns were reported in a study using medical insurance database in China, which showed that during adulthood, the incidence rate of community acquired pneumonia was consistently higher in females than in males until reaching 60 years [5]. The observed sex- and age-specific patterns could be potentially useful for health education and promotion of personal protective behaviours against respiratory infections, although a detailed discussion of the underlying reason for the observed patterns is beyond the scope of this study.

Regarding the hospitalisation cost of ARI, we estimated that the median direct medical cost per ARI episode of older adults was CNY 9027, which was approximately half of what was estimated in our previously published systematic review on ARI management cost in older adults in China (USD 2744 or approximately CNY 19 500) [7]. The main reason for the differences was the length of stay. The MD length of stay in Jiangsu Province from this study was nine days, which was half of the MD length of stay (*i.e.* 18 days) reported in our previous systematic review nationwide. In addition to the length of stay, the cost of ARI episodes varied by region. We found that the GDP per capita was highly positively correlated with the cost of ARI episodes at the municipality level, suggesting higher quality of care in more economically developed regions.

From patients’ perspective, we estimated that the median OOP direct medical cost was CNY 2028, accounting for 22% of the total direct medical cost. The proportion of OOP cost varied by insurance programmes, and was lowest in those covered by UEBMI (MD = 17) that covered employed urban residents, and highest in those covered by NCMS (MD = 40) that covered rural residents. Such urban-rural disparities are concerning when further taking the differences in the disposable income per capita into account (the annual disposable income per capita in Jiangsu Province in 2020 was CNY 60 178 and CNY 28 486 among urban and rural residents, respectively). As a numeric example, even covered by NCMS, the MD OOP cost of an ARI hospitalisation episode of a rural resident was 108% (MD OOP = CNY 2565, yearly disposable income = CNY 28 486) of the monthly disposable income, posing substantial financial burden to the household and equity concerns to the society. This means that rural patients without any forms of health insurance would experience even higher economic burdens from ARI hospitalisation.

As expected, patients with comorbidities generally had higher medical cost of the ARI hospitalisation than those without any comorbidities. However, we showed that the proportion of severe cases in patients with comorbidities was comparable to (stroke and CKD) or even lower (COPD, asthma, diabetes and chronic liver disease) than that in patients without any comorbidities. We speculate that this was due to the varied admission criteria by comorbidity status where ARI patients without any comorbidities were expected to be more severe than those with comorbidities in order to be admitted since comorbidities generally carried high weights in determining the overall severity of pneumonia in common pneumonia severity scoring criteria such as the pneumonia severity index [17].

In China, neither pneumococcal nor influenza vaccines have been included in the national immunisation programme in older adults. These vaccines were only available in the private market and the vaccination coverage was limited. For example, in the 2021–22 season, only 16.6% of adults aged >60 years received influenza vaccination [18]. Since 2023, some regions in China have started to offer free influenza and pneumococcal vaccines to older adults. In Jiangsu Province, Taizhou started a pilot free influenza vaccination programme to adults aged ≥65 years in the 2023–24 season. Therefore, our study provided important baseline data of ARI hospitalisation burden and cost that could be used to evaluate the impact of vaccination programme.

We acknowledge several limitations in this study. First, while the overall medical database was representative of Jiangsu Province, non-grade-III hospitals were less represented in the database. Therefore, the ARI hospitalisation rate in this study was likely to be conservative while severity might be overestimated, and the median cost of ARI episodes could be over-estimated as the medical cost was found lower in non-grade-III hospitals than grade-III hospitals in this study. Moreover, the findings in Jiangsu Province could represent only relatively wealthy provinces of China. Second, as a retrospective analysis of routine medical database, this study was subject to selection and information bias due to varied diagnosis coding practices and hospital information systems. To mitigate the potential bias, we selected only the time period after 2019 for this study when the database and its integration with local hospital information system had been further consolidated. We also implemented additional quality control measures including manual verification of the medical records against the original records; invalid records accounted for less than 0.1% of the total records. Meanwhile, due to the unavailability of relevant data, potential heterogeneities such as regional disparities in admission criteria and health care-seeking behaviour could not be explicitly accounted for. Third, we used ICD-10 coded pathogen-specific diagnosis for comparing different aetiologies of ARI. However, only a small proportion of ARI episodes had a pathogen-specific diagnosis on their clinical records, which could limit the generalisability of the study findings; due to the same reason, we were unable to further compare different specific pathogens (*e.g.* influenza, respiratory syncytial virus, and pneumococcus). Finally, there was no information on the post-discharge outcomes, such as mortality, readmissions and long-term economic impacts; moreover, due to cultural convention, at the end of life, patients prefer to die peacefully at home and would request to be sent home. Therefore, we were likely to have underestimated the true mortality burden of ARI hospitalisation (including the in-hospital mortality burden). Furthermore, we did not assess the longer-term impacts of ARI hospitalisation such as ARI re-admission and post-discharge economic burden.

## CONCLUSIONS

By leveraging a large regionally representative medical database of 0.2 million ARI hospitalisation episodes, with this study, we help address the knowledge gap in the burden and cost of ARI hospitalisation in older adults in China. The overall disease burden and economic burden of ARI in older adults is substantial, with a relatively large proportion of out-of-pocket cost, particularly for rural residents. Several factors such as age, sex, region, and comorbidity status contribute to the variations in the disease burden and cost of ARI. The findings in this study provide important baseline incidence data and have implications for the planning and economic evaluation of relevant vaccination programmes.

## Additional material


Online Supplementary Document
