# Supplementary material for: Incidence and costs of hospitalisation due to acute respiratory infection in adults aged over 50 years in Jiangsu, China in 2019–23: a real-world medical database analysis of 0.2 million episodes
Source: J Glob Health. 2025 Mar 14;15:04116. doi: 10.7189/jogh.15.04116 (PMC11907735; doi:10.7189/jogh.15.04116)
Supplement: Online Supplementary Document [file jogh-15-04116-s001.pdf]

Supplement to: Xu X, Guo L, Li X, Wang X, Li Y. Incidence and costs of hospitalisation due to acute respiratory infection in adults aged over 50 years in Jiangsu, China in 2019–23: a real-world medical database analysis of 0.2 million episodes. J Glob Health. 2025;15:04116.

Table of Contents

Table S1. ICD-10 codes used to identify ARI diagnosis 2
Table S2. Chronic comorbidities and ICD-10 codes (any diagnostic fields)...............3
Table S3. Hospitalisation rates of ARI among those aged 50 years or above in Jiangsu, China (per 1000 person-years) in sensitivity analysis that included the major COVID-19 epidemic period during 11th Dec 2022 to 21st Jan 2023.......................................4
Table S4. Proportion of severe ARI hospitalised patients aged 50 years or above in Jiangsu province (in %) in sensitivity analysis that included the major COVID-19 epidemic period during 11th Dec 2022 to 21st Jan 2023..6
Table S5. Summary of direct medical cost per episode of ARI hospitalisation in CNY of 2023 in sensitivity analysis that included the major COVID-19 epidemic period during 11th Dec 2022 to 21st Jan 2023...7
Table S6**.** Proportion of severe non-COVID ARI hospitalised patients aged 50 years or above in Jiangsu province..10
Table S7. Summary of direct medical cost (Mean ± SD) per episode of non-COVID ARI hospitalisation in CNY of 2023...11
Table S8. Summary of components of direct medical cost per episode of ARI hospitalisation in CNY of 2023 13
Table S9**.** Severity and total direct medical costs of ARI hospitalisation with selected comorbidities vs no comorbidities...14
Figure S1. Sample selection flow chart 15
Figure S2. Hospitalisation rates of non-COVID ARI in different groups by region ..16
Figure S3. Monthly distribution of non-COVID ARI hospitalisations in patients aged 50 years and above in Jiangsu province. The shaded area indicates the time period of the local major COVID-19 epidemic 17
Figure S4. Correlation of GDP per capita with median direct medical costs per episode of ARI among municipalities..18

Table S1. ICD-10 codes used to identify ARI diagnosis.

ICD-10 codes*	Diagnosis
J00-J06	Acute upper respiratory infections
J09-J18	Influenza and pneumonia
J20-J22	Other acute lower respiratory infections

*The ICD-10 codes contain all the sub-codes under their branches, e.g. J01 contains all the codes such as J01.1.

Table S2. Chronic comorbidities and ICD-10 codes (any diagnostic fields).

**ICD-10 codes**	**Diagnosis**
J40, J41.0, J41.1, J41.8, J42.0, J42.1, J42.4, J43.0, J43.1, J43.2, J43.8, J43.9, J44.0, J44.1, J44.8, J44.9, J47.0, J47.1, J47.9	Chronic obstructive pulmonary disease
J45.0, J45.1, J45.2, J45.3, J45.4, J45.5, J45.8, J45.9, J46	Asthma
I20.0, I20.1, I20.8, I20.9, I21.0, I21.1, I21.2, I21.3, I21.4, I21.6, I21.9, I22.0, I22.1, I22.2, I22.8, I22.9, I23.0, I23.1, I23.2, I23.3, I23.4, I23.5, I23.6, I23.7, I23.8, I24.0, I24.1, I24.8, I24.9, I25.0, I25.1, I25.2, I25.3, I25.4, I25.5, I25.6, I25.7, I25.8, I25.9	Ischemic heart disease
I63.0, I63.1, I63.2, I63.3, I63.4, I63.5, I63.6, I63.7, I63.8, I63.9, I65.0, I65.1, I65.2, I65.3, I65.8, I65.9, I66.0, I66.1, I66.2, I66.3, I66.4, I66.8, I66.9, I67.2, I67.3, I67.5, I67.6, I69.3, G45.0, G45.1, G45.2, G45.3, G45.4, G45.8, G45.9, G46.0, G46.1, G46.2, G46.3, G46.4, G46.5, G46.6, G46.7, G46.8.	Stroke
E10.0, E10.1, E10.3, E10.4, E10.5, E10.6, E10.7, E10.8, E10.9, E11.0, E11.1, E11.3, E11.4, E11.5, E11.6, E11.7, E11.8, E11.9, E12.0, E12.1, E12.3, E12.4, E12.5, E12.6, E12.7, E12.8, E12.9, E13.0, E13.1, E13.3, E13.4, E13.5, E13.6, E13.7, E13.8, E13.9, E14.0, E14.1, E14.3, E14.4, E14.5, E14.6, E14.7, E14.8, E14.9, P70.0, P70.1, P70.2, R73.0, R73.9	Diabetes
D63.1, E10.2 , E11.2 , E12.2, E13.2 , E14.2, I12.0, I12.1, I12.2, I12.9, I13.0, I13.1, I13.2, I13.9, N02.0, N02.1, N02.2, N02.3, N02.4, N02.5, N02.6, N02.7, N02.8, N02.9, N03.0, N03.1, N03.2, N03.3, N03.4, N03.5, N03.6, N03.7, N03.8, N03.9, N04.0, N04.1, N04.2, N04.3, N04.4, N04.5, N04.6, N04.7, N04.8, N04.9, N05.0, N05.1, N05.2, N05.3, N05.4, N05.5,N05.6, N05.7, N05.8, N05.9, N06.0, N06.1, N06.2, N06.3, N06.4, N06.5, N06.6, N06.7, N06.8, N06.9, N07.0, N07.1, N07.2, N07.3, N07.4, N07.5, N07.6, N07.7, N07.8, N07.9, N08.0, N08.1, N08.2, N08.3, N08.4, N08.5, N08.8, N15.0, N18.0, N18.1, N18.2, N18.3, N18.4, N18.5, N18.6, N18.8, N18.9, Q61.0 Q61.1, Q61.2, Q61.3, Q61.4, Q61.5, Q61.8, Q61.9, Q62.0, Q62.1, Q62.2, Q62.3 ,Q62.4, Q62.5, Q62.6, Q62.7, Q62.8	Chronic kidney disease
I85.0, I85.9, K70.0, K70.1, K70.2, K70.3, K70.4, K70.9, K71.0, K71.1, K71.3, K71.4, K71.5, K71.7, K71.8, K71.9, K72.0, K72.1, K72.9, K73.0, K73.1, K73.2, K73.8, K73.9, K74.0, K74.1, K74.2, K74.3, K74.4, K74.5, K74.6, K74.9, K75.8, K75.9, K76.0, K76.6, K76.7, K76.9	Chronic liver disease

Table S3. Hospitalisation rates of ARI among those aged 50 years or above in Jiangsu, China (per 1000 person-years) in sensitivity analysis that included the major COVID-19 epidemic period during 11th Dec 2022 to 21st Jan 2023.

Category	Hospitalisation rate (95% CI)				
	Jan. 2019- Dec. 2019	Jan. 2020- Dec. 2020	Jan. 2021- Dec. 2021	Jan. 2022- Dec. 2022	Jun. 2022- May 2023
**All population**	1.07 (1.05, 1.08)	1.09 (1.08, 1.10)	1.46 (1.45, 1.47)	2.19 (2.18, 2.21)	3.32 (3.30, 3.33)
**Age group 50–<60 years**					
All	0.64 (0.63, 0.65)	0.57 (0.56, 0.59)	0.78 (0.77, 0.79)	0.96 (0.94, 0.97)	1.28 (1.26, 1.30)
Male	0.57 (0.56, 0.59)	0.58 (0.56, 0.60)	0.74 (0.73, 0.76)	0.95 (0.93, 0.97)	1.24 (1.21, 1.26)
Female	0.70 (0.68, 0.72)	0.57 (0.55, 0.59)	0.81 (0.79, 0.83)	0.96 (0.94, 0.98)	1.32 (1.29, 1.35)
**Age group 60–<70 years**					
All	0.93 (0.91, 0.95)	0.96 (0.94, 0.98)	1.34 (1.32, 1.36)	1.71 (1.69, 1.74)	2.42 (2.39, 2.45)
Male	1.02 (0.99, 1.04)	1.14 (1.11, 1.17)	1.59 (1.56, 1.63)	2.02 (1.98, 2.06)	2.76 (2.71, 2.80)
Female	0.85 (0.82, 0.87)	0.78 (0.75, 0.80)	1.08 (1.05, 1.11)	1.41 (1.38, 1.44)	2.07 (2.04, 2.11)
**Age group 70–<80 years**					
All	1.41 (1.38, 1.45)	1.59 (1.56, 1.62)	2.12 (2.08, 2.16)	3.36 (3.31, 3.40)	5.30 (5.24, 5.35)
Male	1.72 (1.68, 1.77)	2.07 (2.02, 2.13)	2.76 (2.70, 2.82)	4.36 (4.29, 4.44)	6.76 (6.67, 6.85)
Female	1.12 (1.08, 1.16)	1.14 (1.10, 1.18)	1.51 (1.47, 1.56)	2.40 (2.35, 2.46)	3.91 (3.85, 3.98)
**Age group ≥80 years**					
All	3.09 (3.02, 3.16)	3.16 (3.10, 3.23)	4.07 (4.00, 4.14)	7.53 (7.43, 7.62)	12.30 (12.18, 12.43)
Male	4.32 (4.20, 4.45)	4.77 (4.64, 4.90)	6.14 (6.00, 6.28)	11.22 (11.04, 11.41)	17.75 (17.53, 17.98)
Female	2.20 (2.12, 2.28)	2.03 (1.96, 2.10)	2.56 (2.49, 2.64)	4.79 (4.69, 4.89)	8.26 (8.12, 8.39)
**Region*†**					
Northern Jiangsu	0.85 (0.83, 0.86)	0.81 (0.80, 0.83)	1.07 (1.05, 1.08)	1.75 (1.73, 1.78)	2.83 (2.80, 2.86)
Middle Jiangsu	0.72 (0.70, 0.74)	1.03 (1.00, 1.05)	1.41 (1.38, 1.44)	2.23 (2.19, 2.26)	3.44 (3.40, 3.49)
South Jiangsu	1.37 (1.35, 1.39)	1.33 (1.31, 1.34)	1.79 (1.77, 1.81)	2.51 (2.48, 2.53)	3.62 (3.59, 3.65)

* Northern Jiangsu included five municipalities: Xuzhou, Lianyungang, Suqian, Huaian and Yancheng; Middle Jiangsu included three municipalities: Yangzhou, Taizhou and Nantong; Southern Jiangsu included five municipalities: Nanjing, Suzhou, Wuxi, Changzhou and Zhenjiang.

† The hospitalisation rates of different regions were standardised by age and gender distribution of population census data in Jiangsu Province.

ARI: acute respiratory infection; CI: confidence interval.

Table S4. Proportion of severe ARI hospitalised patients aged 50 years or above in Jiangsu province (in %) in sensitivity analysis that included the major COVID-19 epidemic period during 11th Dec 2022 to 21st Jan 2023.

	Proportion of severe cases* (95% CI)				
**Category**	**Jan. 2019- Dec. 2019**	**Jan. 2020- Dec. 2020**	**Jan. 2021- Dec. 2021**	**Jan. 2022- Dec. 2022**	**Jun. 2022- May 2023**
**All population**	6.54 (6.28, 6.80)	8.05 (7.77, 8.34)	8.18 (7.94, 8.42)	10.93 (10.71, 11.15)	9.20 (9.04, 9.37)
**Age group 50–60 (years)**					
Total	4.29 (3.87, 4.75)	5.78 (5.29, 6.31)	3.55 (3.23, 3.90)	5.30 (4.94, 5.67)	4.60 (4.31, 4.91)
Male	5.84 (5.10, 6.64)	6.50 (5.76, 7.29)	4.83 (4.29, 5.42)	6.61 (6.04, 720)	5.77 (5.31, 6.27)
Female	3.09 (2.61, 3.62)	5.07 (4.42, 5.78)	2.41 (2.05, 2.82)	4.03 (3.59, 4.50)	3.52 (3.17, 3.90)
**Age group 60–70 (years)**					
Total	5.12 (4.69, 5.58)	5.57 (5.12, 6.05)	6.30 (5.89, 6.73)	7.91 (7.52, 8.32)	6.41 (6.11, 6.72)
Male	6.13 (5.50, 6.81)	5.99 (5.38, 6.64)	7.38 (6.81, 7.98)	9.10 (8.56, 9.67)	7.65 (7.22, 8.10)
Female	3.92 (3.37, 4.54)	4.96 (4.29, 5.69)	4.71 (4.15, 5.32)	6.20 (5.66, 6.78)	4.77 (4.37, 5.19)
**Age group 70–80 (years)**					
Total	7.25 (6.70, 7.83)	8.67 (8.10, 9.26)	9.23 (8.74, 9.74)	11.37 (10.96, 11.79)	9.18 (8.88, 9.49)
Male	8.47 (7.70, 9.29)	9.25 (8.52, 10.02)	10.37 (9.72, 11.04)	12.71 (12.16, 13.27)	10.48 (10.08, 10.90)
Female	5.46 (4.71, 6.28)	7.67 (6.80, 8.61)	7.27 (6.55, 8.03)	9.08 (8.47, 9.72)	7.08 (6.65, 7.53)
**Age group ≥80 (years)**					
Total	10.05 (9.38, 10.75)	12.26 (11.57, 12.96)	13.73 (13.12, 14.36)	16.46 (15.98, 16.95)	13.52 (13.18, 13.87)
Male	11.51 (10.60, 12.48)	12.99 (12.10, 13.91)	15.45 (14.64, 16.28)	19.11 (18.47, 19.76)	16.24 (15.76, 16.72)
Female	7.95 (7.03, 8.96)	11.04 (9.98, 12.16)	10.75 (9.84, 11.71)	11.88 (11.19, 12.59)	9.21 ((8.74, 9.69)

*Defined as the use of mechanical ventilation, admission to intensive care unit (ICU), or death.

ARI: acute respiratory infection; CI: confidence interval.

Table S5. Summary of direct medical cost per episode of ARI hospitalisation in CNY of 2023 in sensitivity analysis that included the major COVID-19 epidemic period during 11th Dec 2022 to 21st Jan 2023.

Category	N	Total cost Median (IQR)	OOP cost Median (IQR)	Proportion of OOP cost (%) Median (IQR)	Length of stay Median (IQR)
**All population**	259,122	9,037 (6,073- 14,617)	2,036 (583- 4,116)	22 (7- 42)	9 (6- 12)
**Age group (years)**					
50-<60	53,205	7,275 (5,075- 10,635)	1,777 (439- 3,385)	25 (7- 44)	8 (6- 11)
60-<70	62,728	8,416 (5,799- 12,824)	1,932 (543- 3,805)	23 (7- 42)	8 (6- 12)
70-<80	72,026	9,505 (6,462- 15,396)	2,187 (696- 4,376)	22 (8- 42)	9 (6- 13)
≥80	71,163	11,213 (7,193- 20,488)	2,241 (619- 4,817)	20 (5- 39)	10 (7- 15)
**Sex**					
Male	149,861	9,767 (6,406- 16,786)	2,006 (515- 4,338)	20 (6- 39)	9 (6- 13)
Female	109,146	8,246 (5,715- 12,365)	2,071 (687- 3,884)	25 (9- 45)	8 (6- 11)
**Grade of Hospital**					
Grade III general hospital	218,139	9,143 (6,164- 14,856)	2,077 (594- 4,187)	22 (7- 42)	9 (6- 12)
Grade III specialty hospital	24,109	10,036 (6,822- 15,731)	2,040 (548- 4,284)	19 (6- 35)	10 (7- 14)
Grade II hospital	16,716	6,695 (4,708- 9,943)	1,617 (532- 3,016)	25 (9- 43)	7 (5- 10)
Other medical services	158	3,649 (2,641- 5,165)	569 (4- 923)	16 (0- 27)	7 (6- 9)
**Disease Category**					
Pneumonia and Influenza	217,035	9,709 (6,607- 15,976)	2,212 (667- 4,495)	22 (7- 41)	9 (7- 13)
AURI	15,367	4,551 (3,201- 6,730)	1,216 (240- 2,213)	26 (6- 49)	5 (4- 8)
Other ALRI	26,720	7,149 (5,054- 10,120)	1,527 (384- 2,824)	22 (5- 41)	7 (5- 10)
**Time Period**					
Jan. 2019- Dec. 2019	33,749	8,705 (5,823- 13,670)	1,838 (362- 3,722)	21 (5- 41)	8 (6 -12)
Jan. 2020- Dec. 31st, 2020	35,486	9,330 (6,197- 15,420)	1,864 (470- 3,922)	20 (6- 39)	9 (6- 13)

Jan. 2021- Dec. 2021	49,674	9,485 (6,372- 15,570)	2,068 (635- 4,254)	21 (7- 39)	9 (6- 13)
Jan. 2022- Dec. 2022	76,892	9,014 (6,038- 14,974)	2,132 (711- 4,346)	23 (8- 43)	9 (6- 13)
Jun. 2022- May 2023	115,857	8,887 (5,994- 14,338)	2,100 (644- 4,177)	23 (8- 43)	9 (6- 13)
**ICU admission**					
Yes	17,431	33,956 (10,472- 77,905)	5,098 (1,365- 15,351)	19 (8- 37)	11 (6- 19)
No	241,691	8,766 (5,975- 13,529)	1,965 (554- 3,844)	22 (7- 42)	9 (6- 12)
**Clinical Outcome**					
Fully recovered	11,003	6,102 (4,071- 9,622)	1,372 (171- 2,778)	22 (3- 42)	7 (5- 10)
Improved	228,121	8,948 (6,147- 13,864)	2,018 (586- 3,946)	22 (7- 42)	9 (6- 12)
Not improved	13,161	17,574 (7,457- 48,050)	3,449 (1,028- 10,362)	23 (10- 43)	7 (3- 13)
Died	4,127	34,737 (13,673- 80,647)	4,294 (599- 14,044)	17 (3- 29)	9 (4- 17)
Unknown	2,710	8,777 (5,004- 21,040)	2,391 (651- 6,017)	24 (8- 55)	7 (3- 12)
**Source of admission**					
Emergency	90,775	10,276 (6,532- 19,359)	2,430 (900- 5,193)	23 (10- 42)	9 (6- 13)
Outpatient	165,363	8,549 (5,884- 12,989)	1,849 (433- 3,646)	22 (5- 41)	9 (6- 12)
Transfer	1,950	8,352 (5,575- 13,493)	2,096 (667- 4,794)	23 (9- 49)	8 (6- 12)
Other	1,034	8,957 (5,836- 18,616)	2,630 (783- 5,912)	20 (8- 100)	9 (6- 13)
**Ventilation Use Time (hours)**					
0	254,339	8,923 (6,028- 14,185)	2,000 (558- 3,987)	22 (7- 42)	9 (6- 12)
1-96	1,846	24,416 (12,133- 45,099)	5,711 (2,281- 13,048)	26 (14- 43)	5 (2- 12)
>96	2,937	90,977 (55,756- 146,950)	16,878 (7,836- 32,745)	19 (11- 32)	18 (11- 28)
**Antibiotics Use**					
Yes	191,172	9,325 (6,339- 15,024)	2,222 (833- 4,343)	23 (10- 42)	9 (6- 13)
No	62,797	8,076 (5,296- 13,352)	1,463 (10- 3,285)	19 (0- 41)	8 (6- 12)

**Pathogen-specific ICD diagnosis**					
Bacterial infection	1,538	24,681 (13,121- 49,880)	3,543 (1,241- 7,761)	15 (5- 26)	18 (11- 26)
Viral infection	1,159	7,396 (4,949- 11,611)	2,048 (891- 3,874)	29 (12- 47)	7 (5- 10)
**Health insurance**					
UEBMI	131,289	9,539 (6,428- 15,563)	1,618 (295- 3,213)	17 (3- 27)	9 (6- 13)
URBMI	85,835	8,171 (5,641- 12,358)	2,497 (972- 4,439)	35 (13- 49)	8 (6- 11)
NCMS	9,097	7,515 (5,189- 11,200)	2,565 (1,235- 4,176)	40 (18- 49)	8 (6- 11)
Others	32,901	10,605 (6,402- 20,170)	3,086 (765- 8,067)	28 (6- 100)	9 (6- 14)
**Region***					
Northern Jiangsu	73,061	7,302 (5,072- 10,744)	1,979 (790- 3,474)	28 (11- 46)	8 (6- 11)
Middle Jiangsu	49,997	8,991 (6,066- 14,034)	1,968 (479- 4,433)	22 (5- 46)	9 (6- 12)
Southern Jiangsu	135,444	10,306 (6,876- 17,400)	2,097 (422- 4,494)	20 (5- 36)	9 (7- 13)

* Northern Jiangsu included five municipalities: Xuzhou, Lianyungang, Suqian, Huaian and Yancheng; Middle Jiangsu included three municipalities: Yangzhou, Taizhou and Nantong; Southern Jiangsu included five municipalities: Nanjing, Suzhou, Wuxi, Changzhou and Zhenjiang.

ARI: acute respiratory infection; CNY: Chinese Yuan; IQR: interquartile range; OOP: out of pocket; AURI: acute upper respiratory infection; ALRI: acute lower respiratory infection; UEBMI: Urban Employee Basic Medical Insurance; URBMI: Urban Resident Basic Medical Insurance; NCMS: New Rural Cooperative Medical Scheme.

Table S6. Proportion of severe non-COVID ARI hospitalised patients aged 50 years or above in Jiangsu province.

Category	Proportion of severe cases*, % (95% CI)				
	**Jan. 2019- Dec. 2019**	**Jan. 2020- Dec. 2020**	**Jan. 2021- Dec. 2021**	**Jan. 2022- Dec. 2022**	**Jun. 2022- May. 2023**
**All population**	6.54 (6.28, 6.80)	8.05 (7.77, 8.34)	8.18 (7.94, 8.42)	10.28 (10.02, 10.53)	8.75 (8.54, 8.97)
**Age group 50–<60 (years)**					
Total	4.29 (3.87, 4.75)	5.78 (5.29, 6.31)	3.55 (3.23, 3.90)	5.33 (4.92. 5.75)	4.87 (4.51, 5.25)
Male	5.84 (5.10, 6.64)	6.50 (5.76, 7.29)	4.83 (4.29, 5.42)	6.65 (6.01, 7.33)	5.98 (5.41, 6.59)
Female	3.09 (2.61, 3.62)	5.07 (4.42, 5.78)	2.41 (2.05, 2.82)	4.05 (3.56, 4.59)	3.83 (3.38, 4.32)
**Age group 60–<70 (years)**					
Total	5.12 (4.69, 5.58)	5.57 (5.12, 6.05)	6.30 (5.89, 6.73)	7.87 (742, 8.34)	6.46 (6.08, 6.85)
Male	6.13 (5.50, 6.81)	5.99 (5.38, 6.64)	7.38 (6.81, 7.98)	8.97 (8.36, 9.62)	7.58 (7.05, 8.14)
Female	3.92 (3.37, 4.54)	4.96 (4.29, 5.69)	4.71 (4.15, 5.32)	6.25 (5.62, 6.92)	4.92 (4.41, 5.47)
**Age group 70–<80 (years)**					
Total	7.25 (6.70, 7.83)	8.67 (8.10, 9.26)	9.23 (8.74, 9.74)	11.03 (10.54, 11.53)	9.28 (8.88, 9.71)
Male	8.47 (7.70, 9.29)	9.25 (8.52, 10.02)	10.37 (9.72, 11.04)	12.01 (1138, 12.67)	10.09 (9.55, 10.64)
Female	5.46 (4.71, 6.28)	7.67 (6.80, 8.61)	7.27 (6.55, 8.03)	9.33 (8.59, 10.11)	7.95 (7.32, 8.61)
**Age group ≥80 (years)**					
Total	10.05 (9.38, 10.75)	12.26 (11.57, 12.96)	13.73 (13.12, 14.36)	15.47 (14.90, 16.06)	13.29 (12.78, 13.80)
Male	11.51 (10.60, 12.48)	12.99 (12.10, 13.91)	15.45 (14.64, 16.28)	17.63 (16.88, 18.41)	15.70 (15.01, 16.40)
Female	7.95 (7.03, 8.96)	11.04 (9.98, 12.16)	10.75 (9.84, 11.71)	11.61 (10.76, 12.49)	9.30 (8.60, 10.04)

*Defined as the use of mechanical ventilation, admission to intensive care unit (ICU), or death.

COVID: coronavirus disease; ARI: acute respiratory infection; CI: confidence interval.

Table S7. Summary of direct medical cost (Mean ± SD) per episode of non-COVID ARI hospitalisation in CNY of 2023.

Category	Total cost Mean ± SD
**All population**	16,488 ± 27,120
**Age group (years)**	
50-<60	11,122 ± 18,836
60-<70	13,893 ± 22,282
70-<80	17,637 ± 28,232
≥80	22,853 ± 34,514
**Sex**	
Male	18,873 ± 30,519
Female	13,214 ± 21,171
**Grade of Hospital**	
Grade III general hospital	16,845 ± 27,668
Grade III specialty hospital	17,776 ± 27,945
Grade II hospital	10,432 ± 16,330
Other medical services	3,740 ± 1,787
**Disease Category**	
Pneumonia and Influenza	18,445 ± 29,434
AURI	5,954 ± 5,637
Other ALRI	9,458 ± 11,381
**Time Period**	
Jan. 2019- Dec. 2019	14,832 ± 24,148
Jan. 2020- Dec. 2020	16,975 ± 27,340
Jan. 2021- Dec. 2021	17,489 ± 28,198
Jan. 2022- Dec. 2022	17,663 ± 29,883
Jun. 2022- May. 2023	16,122 ± 26,950
**ICU admission**	
Yes	55,376 ± 62,253
No	13,578 ± 19,467
**Clinical Outcome**	
Fully recovered	10,384 ± 18,509
Improved	14,888 ± 22,854
Not improved	41,850 ± 54,043
Died	68,418 ± 69,687
Unknown	22,262 ± 39,679
**Source of admission**	
Emergency	22,304 ± 35,255
Outpatient	13,580 ± 21,198
Transfer	16,279 ± 30,789
Other	25,211 ± 46,659
**Ventilation Use Time (hours)**	
0	15,375 ± 24,280

1-96	40,081 ± 41,429
>96	111,913 ± 70,443
**Antibiotics Use**	
Yes	16,935 ± 27,539
No	15,162 ± 25,966
**Pathogen-specific ICD diagnosis**	
Bacterial infection	40,702 ± 45,983
Viral infection	11,701 ± 18,687
**Health insurance**	
UEBMI	17,778 ± 29,031
URBMI	12,970 ± 19,742
NCMS	10,783 ± 14,434
Others	22,521 ± 36,200
**Region***	
Northern Jiangsu	11,437 ± 18,757
Middle Jiangsu	15,619 ± 25,658
Southern Jiangsu	19,446 ± 30,639

* Northern Jiangsu included five municipalities: Xuzhou, Lianyungang, Suqian, Huaian and Yancheng; Middle Jiangsu included three municipalities: Yangzhou, Taizhou and Nantong; Southern Jiangsu included five municipalities: Nanjing, Suzhou, Wuxi, Changzhou and Zhenjiang.

COVID: coronavirus disease; ARI: acute respiratory infection; CNY: Chinese Yuan; SD: standard deviation; AURI: acute upper respiratory infection; ALRI: acute lower respiratory infection; UEBMI: Urban Employee Basic Medical Insurance; URBMI: Urban Resident Basic Medical Insurance; NCMS: New Rural Cooperative Medical Scheme.

Table S8. Summary of components of direct medical cost per episode of ARI hospitalisation in CNY of 2023.

Cost category	Total Cost Median (IQR)	Percentage of the total cost (%)
**Nursing cost**	283 (182- 473)	3 (2- 4)
**Diagnosis cost**	3,437 (2,252- 5,201)	37 (26- 49)
Laboratory diagnosis cost	2,401 (1,537- 3,620)	25 (17- 35)
Imaging diagnosis cost	545 (225- 1,060)	5 (2- 10)
Clinical diagnosis cost	111 (34- 546)	1 (0- 5)
**Treatment cost**	18 (0- 389)	0 (0- 4)
**Medicine cost**	3,289 (1,831- 5,947)	37 (26- 47)
**Consumable cost**	275 (118- 664)	3 (2- 6)

IQR: interquartile range; ARI: acute respiratory infection; CNY: Chinese Yuan.

Table S9. Severity and total direct medical costs of ARI hospitalisation with selected comorbidities vs no comorbidities.

Comorbidity	N	Proportion of severity*, % (95% CI)	Total cost† Median (IQR)
COPD	17,358	4.78 (4.47- 5.11)	9,379 (6,723- 14,003)
Asthma	2,832	2.65 (2.09- 3.31)	9,207 (6,901- 12,308)
IHD	14,262	4.35 (4.03- 4.70)	9,018 (6,416- 13,235)
Stroke	12,799	6.81 (6.37- 7.26)	9,904 (6,605- 16,861)
Diabetes	17,057	3.65 (3.38- 3.94)	8,733 (6,085- 12,864)
CKD	2,099	8.05 (6.92- 9.30)	10,102 (6,777- 16,385)
CLD	6,475	4.56 (4.06- 5.09)	8,601 (6,232- 12,270)
No comobidities	18,482	7.16 (6.80- 7.55)	6,085 (4,237- 8,731)

Comorbidity status was based on diagnosis information shown in the discharge diagnosis field.

* Defined as the use of mechanical ventilation, admission to intensive care unit (ICU), or death.

†The costs were reported in CNY of 2023.

ARI: acute respiratory infection; CI: confidence interval; IQR: interquartile range; COPD: chronic obstructive pulmonary disease; IHD: ischemic heart disease; CKD: chronic kidney disease; CLD: chronic liver disease.

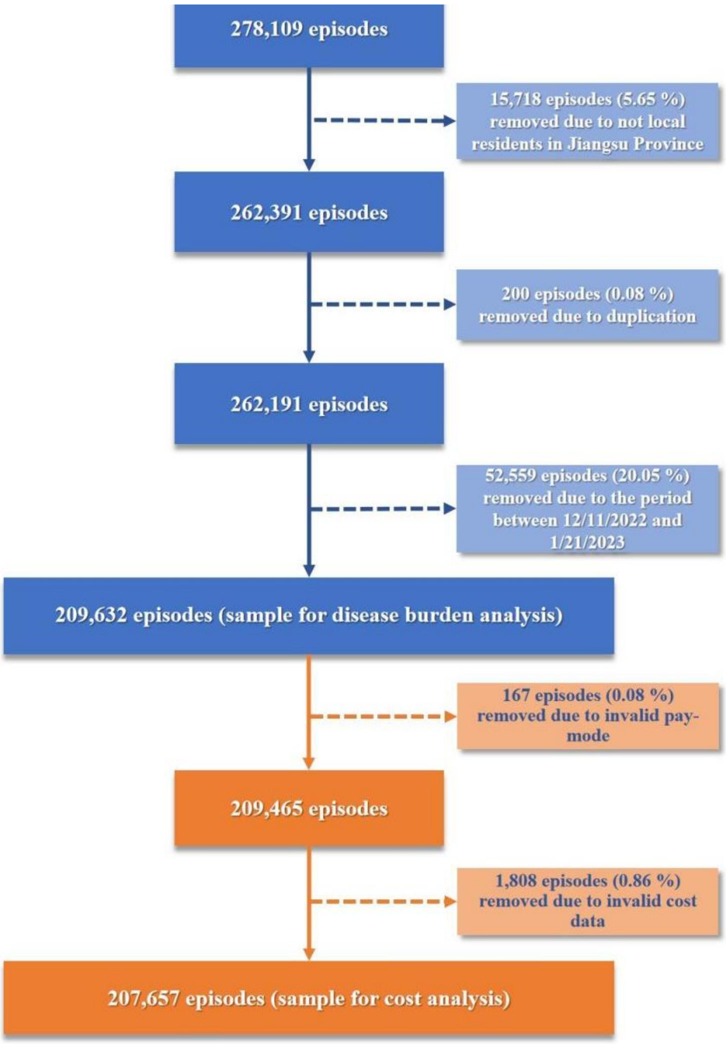

Figure S1. Sample selection flow chart.

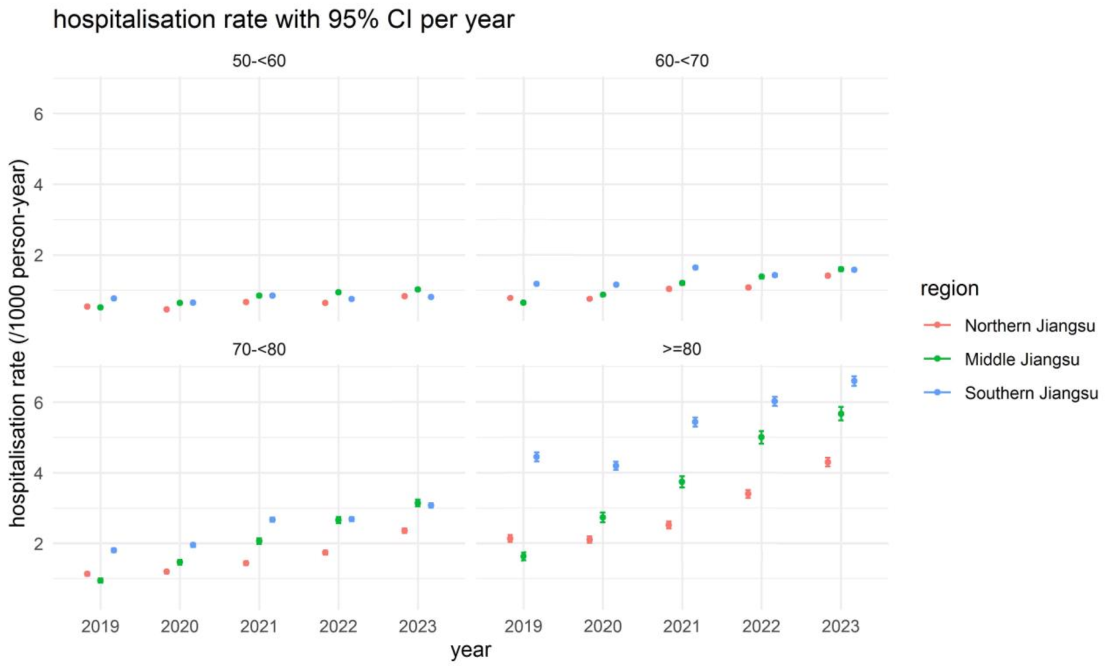

Figure S2. Hospitalisation rates of non-COVID ARI in different groups by region.

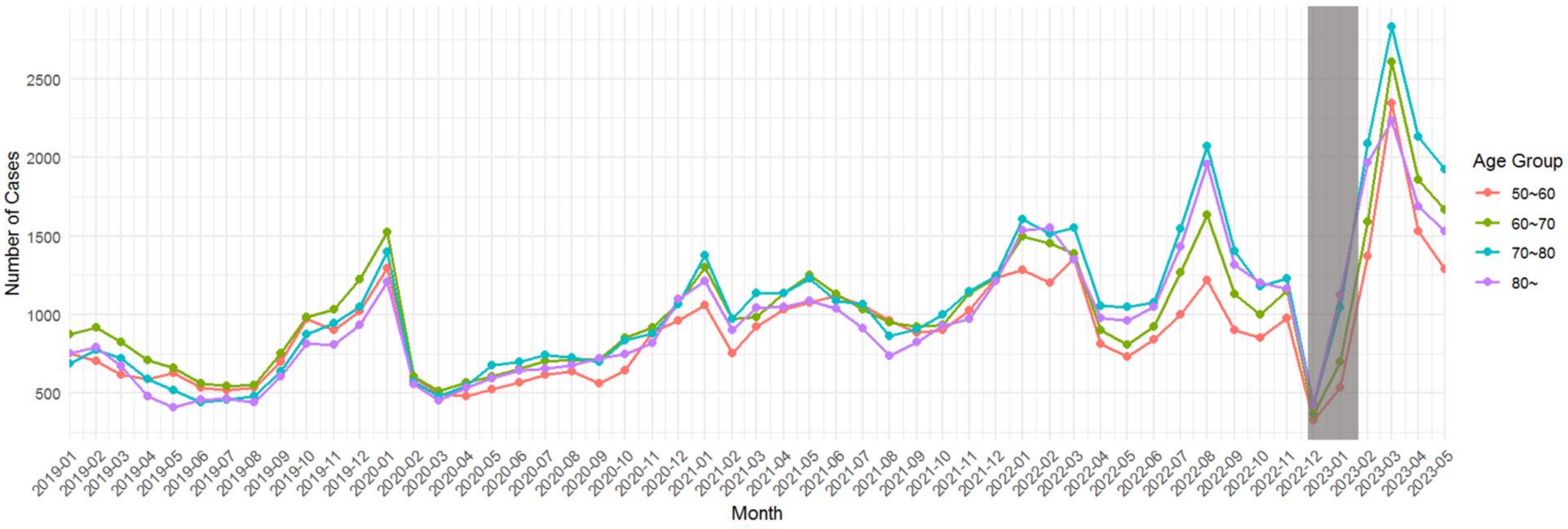

Figure S3. Monthly distribution of non-COVID ARI hospitalisations in patients aged 50 years and above in Jiangsu province. The shaded area indicates the time period of the local major COVID-19 epidemic.

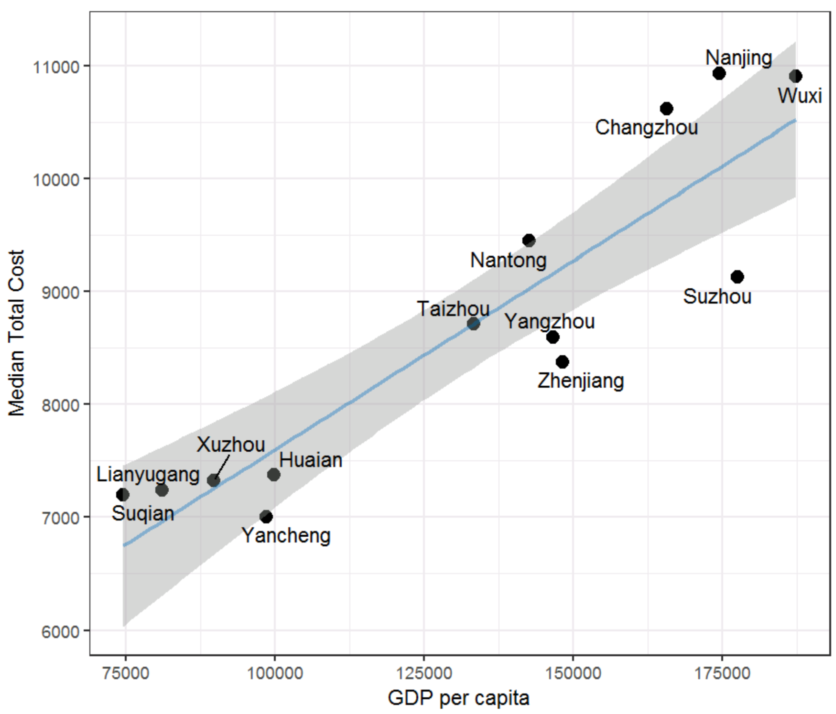

Figure S4. Correlation of GDP per capita with median direct medical costs per episode of ARI among municipalities.

The dots represent GDP per capita (horizontal) and the median cost (vertical). The blue line represents the linear correlation. Linear correlation coefficient r = 0.907. GDP: gross domestic product; ARI: acute respiratory infection.
